# Anesthetic Implications for Patients With Implanted Hypoglossal Nerve Stimulators: A Case Report

**DOI:** 10.7759/cureus.21424

**Published:** 2022-01-19

**Authors:** Jose R Soberon, Irina Murray Casanova, Jonathan Wright

**Affiliations:** 1 Anesthesiology, North Florida/Southern Georgia Veterans Health System, Gainesville, USA; 2 Anesthesiology, University of Florida, Gainesville, USA; 3 Anesthesiology, University of Central Florida, Orlando, USA; 4 Surgery, North Florida/Southern Georgia Veterans Health System, Gainesville, USA; 5 Surgery, University of Florida, Gainesville, USA; 6 Orthopaedics and Rehabilitation Surgery, North Florida/Southern Georgia Veterans Health System, Gainesville, USA; 7 Orthopaedics and Rehabilitation Surgery, University of Florida, Gainesville, USA

**Keywords:** implantable medical device, perioperative evaluation, central alveolar hypoventilation, continuous positive airway pressure (cpap), alveolar hypoventilation, hypoglossal nerve stimulator, obstructive sleep apnea (osa)

## Abstract

Obstructive sleep apnea is a serious health issue affecting more than one billion people worldwide. Although continuous positive airway pressure is the mainstay for the treatment of obstructive sleep apnea, hypoglossal nerve stimulation is a surgical option for patients who are unable to tolerate or adhere to this therapy. As more hypoglossal nerve stimulators are implanted, these patients will present with increasing frequency for medical procedures requiring general anesthesia or deep sedation. We describe our experience with one such patient and hope this information can be used to develop future guidelines to aid in the anesthetic management of these patients.

## Introduction

Obstructive sleep apnea (OSA) affects over one billion people worldwide, including approximately 22 million Americans [1,2]. By maintaining airway patency during sleep, the regular use of continuous positive airway pressure (CPAP) improves the quality of life, vigilance, and cognitive function in patients with OSA [1]. Despite these favorable outcomes, only 40-60% of patients with OSA are compliant with CPAP therapy [2].

Hypoglossal nerve stimulation (HGNS) is a surgical option for patients with moderate to severe OSA who have failed or are nonadherent to CPAP therapy [2-4]. Inspire Medical Systems, Inc. (Golden Valley, MN, USA) offers a surgically implantable system to treat OSA, which was approved by the United States Food and Drug Administration in 2014. This system consists of a stimulating electrode encircling the right hypoglossal nerve, a pressure-sensing lead, and an implantable pulse generator [2,3]. Since the hypoglossal nerve innervates the majority of the intrinsic and extrinsic muscles of the tongue [2], stimulation of this nerve increases airway tone and diameter to promote patency of the upper airway [2,3]. Unlike other surgical procedures for OSA (e.g., uvulopalatopharyngoplasty), the HGNS system does not modify oropharyngeal structures [3]. The majority of patients who receive the HGNS system report improvement in OSA symptoms and quality of life, and prefer it over CPAP therapy [2-4].

Once programmed, settings for the HGNS system are stored in the device generator, and a sleep remote is used by the patient to turn the device on before sleep (and off once awake). The device detects respiratory efforts and delivers mild electrical stimulation to the hypoglossal nerve, increasing or maintaining airway patency via tongue protrusion. 

As the implantation of these devices becomes increasingly common, more patients with HGNS systems will be present for medical procedures requiring general anesthesia or deep sedation. Currently, guidelines for the anesthetic management of OSA do not address patients with HGNS systems [1,5]. Furthermore, a search of the published literature produced no citations or similar case presentations concerning anesthesia for patients with HGNS systems. We report a case of a patient with the HGNS system undergoing shoulder surgery in the beach chair position to bring awareness to these devices and encourage further research in the management of these patients.

The patient provided written Health Insurance Portability and Accountability Act (HIPAA) authorization to publish this case report.

## Case presentation

**Figure 1:**
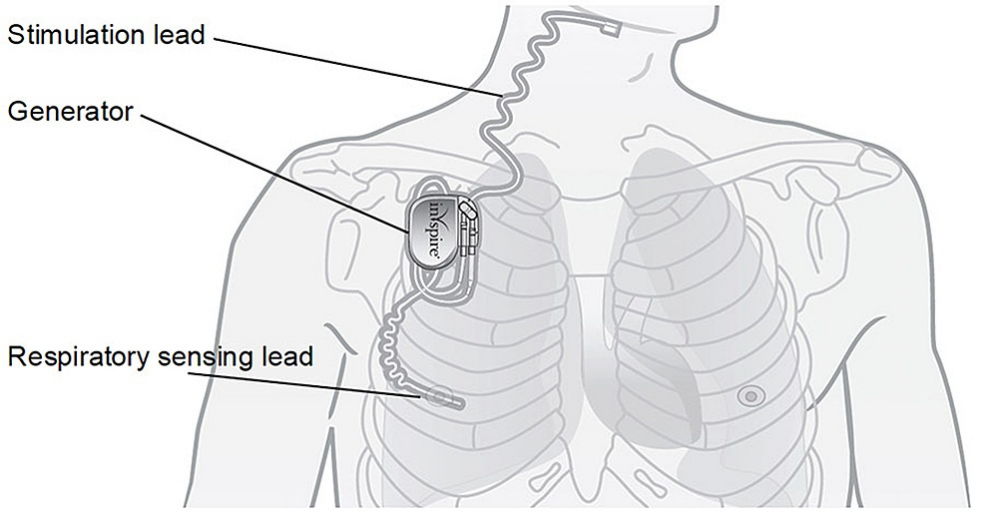
Schematic of the implanted HGNS system. The system consists of a stimulating electrode, pressure-sensing lead, and a pulse generator. Reused with permission of Inspire Medical Systems, Inc. HGNS: hypoglossal nerve stimulation.

**Figure 2:**
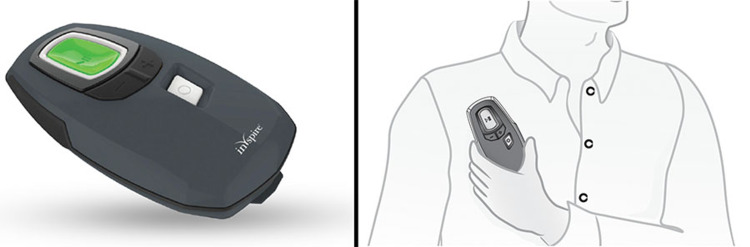
Sleep remote for the HGNS system. Reused with permission of Inspire Medical Systems, Inc. HGNS: hypoglossal nerve stimulation.

A 53-year-old, 109-kg male bodybuilder with a history of hypertension, polycythemia, and daily marijuana use (American Society of Anesthesiologists Physical Status II) presented for total shoulder arthroplasty. Physical examination revealed a muscular physique with a thick neck but otherwise favorable airway characteristics. During the preoperative interview, he stated that he had once been diagnosed with severe OSA but was “cured” by the placement of the HGNS system. The device was placed at an outside hospital per the recommendation of a pulmonologist three years prior. He reported being satisfied with the device and would activate it every evening prior to bedtime and would deactivate it after awakening in the morning. He did not bring the device card or remote control before surgery. Figure 1 and Figure 2 depict the system.

Ultrasound-guided single-injection interscalene and superficial cervical plexus blocks were successfully performed prior to surgery. Upon arrival to the operating room, continuous pulse oximetry, electrocardiographic monitoring, and non-invasive blood pressure measurements were instituted. Baseline vital signs (prior to any surgical or anesthetic intervention) were blood pressure 144/96, heart rate 73, SpO_2_ 99% on room air, and a respiratory rate of 16. A non-invasive nasal positive pressure mask (SuperNO2VA^TM^ mask, Vyaire Medical, Mettawa, IL, USA) was placed prior to the administration of procedural sedation. A propofol infusion was started at 100 mcg/kg/min and ketamine was incrementally administered intravenously in 10-20 mg increments (concentration 10 mg/mL) until a state of deep sedation was achieved. The propofol infusion was then decreased to 50 mcg/kg/min and the patient was placed in a beach chair position.

Once the surgical procedure was underway, the patient experienced episodes of airway obstruction with pulse oximetry values of 88% that persisted despite adjustments to the nasal CPAP mask. A nasal trumpet was placed and the propofol infusion was temporarily halted, which improved ventilation and oxygenation. Additional doses of ketamine were administered intravenously, and the propofol infusion was subsequently resumed at a lower rate to preserve airway tone. The surgical procedure was described as unexpectedly difficult and had a duration of approximately 210 minutes. A total of 200 mg of ketamine (1.83 mg/kg) was administered, with the last dose given over two hours prior to wound closure. No benzodiazepines, narcotics, sedatives, or other analgesics were administered during surgery.

The patient was transported to the postanesthesia care unit and was found to be unarousable. His pupils were sluggishly reactive, but there was no motor, verbal, or eye response to voice or noxious stimuli. Initial laboratory testing yielded the following results: blood glucose 197, arterial pH 7.34, arterial pO_2_ 122 mm Hg, and arterial pCO_2_ 43 mm Hg. The patient also experienced episodes of airway obstruction and oxygen desaturation whenever the nasal CPAP mask was removed. At that time, the last ketamine dose had been four hours earlier, and the propofol infusion had been discontinued an hour earlier. Because of his history of hypertension and polycythemia (which places him at an elevated risk of thromboembolic events [6]), an urgent neurologic consult was requested. He was immediately intubated and transferred to the surgical intensive care unit (ICU) for further testing and management. 

**Figure 3:**
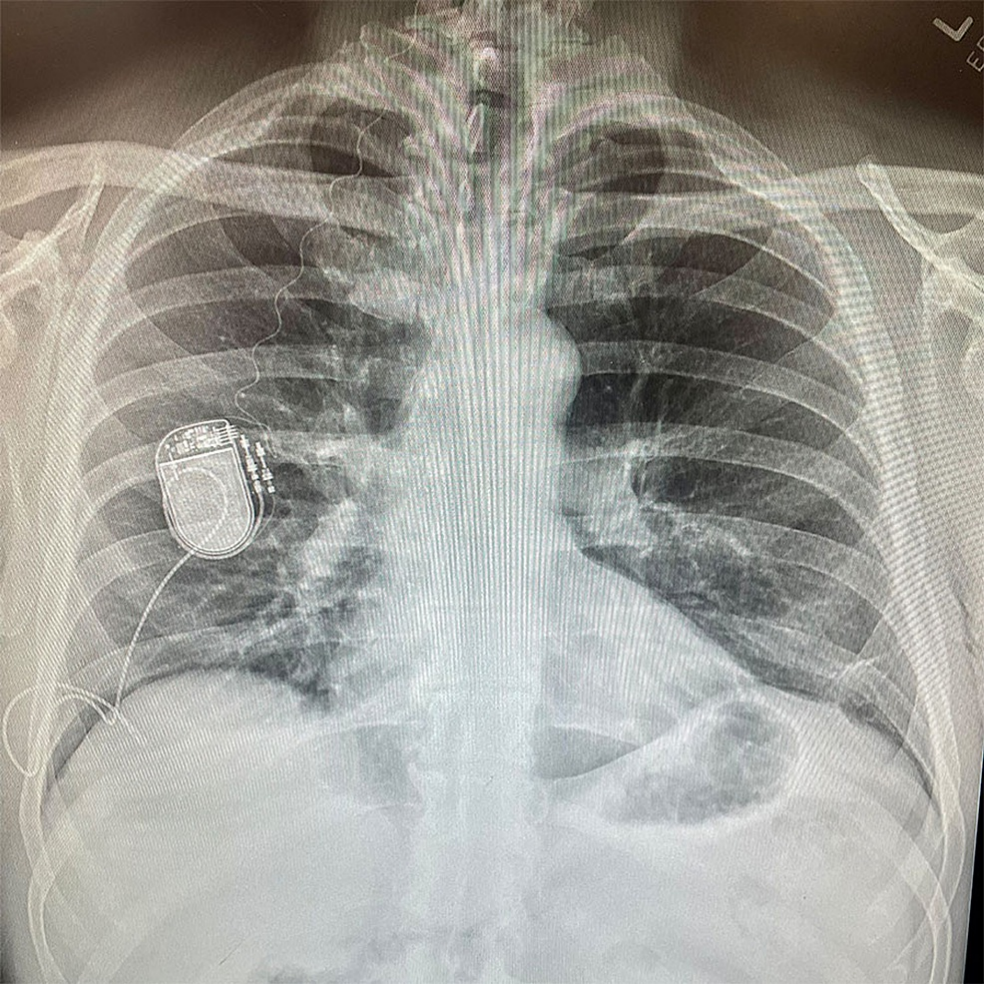
Chest radiograph showing the HGNS system. HGNS: hypoglossal nerve stimulation.

Additional laboratory testing and emergent head CT scans were unremarkable. A chest x-ray performed in the ICU found “a radiopaque monitoring device with leads extending both superiorly and inferiorly from the battery pack” (Figure 3). He spontaneously regained consciousness approximately three hours later and was subsequently extubated. Neurologic examination was normal, and he was discharged home the following day. 

## Discussion

At the time of this writing, there are no published citations regarding the management of HGNS systems in patients undergoing procedural sedation or general anesthesia. Furthermore, these devices are not mentioned in recent articles pertaining to OSA in the perioperative period [1,5]. We contacted Inspire Medical Systems and were informed that approximately 17,000 patients in the United States have had a HGNS system implanted. Furthermore, the manufacturer stated (written communications to JRS, September 30, 2021) that the “use of Inspire for airway maintenance outside of our indication (OSA) is off label so we cannot offer any guidance(.) . . . Inspire therapy is not intended for life-sustaining airway management. If ventilation support is needed for the patient during recovery, utilize appropriate equipment such as CPAP. Do not use Inspire therapy for life-sustaining airway maintenance”.

The role of an HGNS system during airway management is unknown. While stimulation of the hypoglossal nerve may be beneficial during deep sedation or recovery from anesthesia, its use may adversely affect supraglottic tube placement and potentially risk dislodgement of the supraglottic device in the absence of neuromuscular blocking agents. Its effects on mask ventilation and intubating conditions are similarly unknown. There are no manufacturer recommendations for perioperative management of these devices at this time. 

Inquiring about the presence of HGNS systems should be included in the preoperative evaluation as is similarly done with pacemakers, defibrillators, spinal cord stimulators, and other devices. In the aforementioned case, it is unknown whether activating the device would have improved airway patency intra- and postoperatively. Because the patient did not bring his remote control, activating the device was not possible during his episodes of airway obstruction. Due to a lack of experience or established protocols, we were unable to ask our patient to bring his remote control with him to the procedure. The remote control would have given us the option to utilize his hypoglossal nerve stimulator and potentially ameliorate upper airway obstruction. 

In the absence of manufacturer recommendations or evidence-based guidelines, we would recommend inquiring about hypoglossal nerve stimulators during the preoperative evaluation and, if possible, asking the patient to bring their device remote on the date of their procedure. If the anesthetic plan is deep sedation, our choice would be to activate the device at the commencement of sedation administration and deactivate it once the patient has regained consciousness. If a general anesthetic is planned, we suggest not activating the device during airway management if neuromuscular blockers are not utilized to minimize tongue protrusion, activating it once removal of the airway device has occurred, and then deactivating it once the patient has sufficiently emerged from the general anesthetic.

## Conclusions

Implantation of HGNS systems is increasingly used as an alternative to CPAP therapy, and these patients will likely be present for other medical procedures as well. Patients with HGNS systems should be presumed to have moderate to severe OSA and this should be taken into consideration when formulating an anesthetic plan. A HGNS system should be added to the differential diagnosis of implantable devices that can be detected during routine perioperative imaging. Further research is necessary to determine the safety and efficacy of HGNS use during the perioperative period as well as to facilitate the creation of guidelines to manage and identify devices. 

## References

[1] Cozowicz C, Memtsoudis SG (2021). Perioperative management of the patient with obstructive sleep apnea: a narrative review. Anesth Analg.

[2] Yu JL, Thaler ER (2020). Hypoglossal nerve (cranial nerve XII) stimulation. Otolaryngol Clin North Am.

[3] Woodson BT, Strohl KP, Soose RJ (2018). Upper airway stimulation for obstructive sleep apnea: 5-year outcomes. Otolaryngol Head Neck Surg.

[4] Costantino A, Rinaldi V, Moffa A (2020). Hypoglossal nerve stimulation long-term clinical outcomes: a systematic review and meta-analysis. Sleep Breath.

[5] Memtsoudis SG, Cozowicz C, Nagappa M (2018). Society of anesthesia and sleep medicine guideline on intraoperative management of adult patients with obstructive sleep apnea. Anesth Analg.

[6] Marchioli R, Finazzi G, Landolfi R (2005). Vascular and neoplastic risk in a large cohort of patients with polycythemia vera. J Clin Oncol.

